# Double-Stranded RNA Mycovirus Infection of *Aspergillus fumigatus* Is Not Dependent on the Genetic Make-Up of the Host

**DOI:** 10.1371/journal.pone.0077381

**Published:** 2013-10-22

**Authors:** Jeannine M. Refos, Alieke G. Vonk, Kimberly Eadie, Jerome R. Lo-Ten-Foe, Henri A. Verbrugh, Anne D. van Diepeningen, Wendy W. J. van de Sande

**Affiliations:** 1 Department of Medical Microbiology and Infectious Diseases, Erasmus MC, Rotterdam, The Netherlands; 2 Department of Medical Microbiology, University Medical Center Groningen, University of Groningen, Groningen, The Netherlands; 3 Fungal Biodiversity Centre, Utrecht, The Netherlands; Leibniz Institute for Natural Products Research and Infection Biology- Hans Knoell Institute, Germany

## Abstract

*Aspergillus fumigatus* is a fungus that causes opportunistic infections in immunocompromised patients, with high morbidity and mortality. In its turn, *A. fumigatus* can become infected with mycoviruses. Most mycoviruses have a dsRNA genome and can cause fungal hypovirulence. For that reason, mycoviruses could theoretically be used as therapeutic tools to combat fungal infections. We determined if a certain genetic make-up of *A. fumigatus* was associated with the presence of mycoviruses in 86 clinical *A. fumigatus* isolates. Mycovirus screening was performed by isolating dsRNA from mycelial cultures using a Trizol/Chloroform method. The genetic relatedness of dsRNA infected *A. fumigatus* was determined by cell surface protein (CSP) typing and determination of the mating type. Sixteen (18.6%) of the 86 clinical *A. fumigatus* isolates contained dsRNA. The *A. fumigatus* collection could be divided into 11 different CSP types. DsRNA infected *A. fumigatus* isolates had similar CSP types as non-infected isolates. In both cases, the CSP types t01, t02, t03 and t04 were the most prevalent and the distribution comparable to the CSP types observed in other Dutch collections. Mating types MAT1-1 and MAT1-2 were evenly distributed among all *A. fumigatus* strains, regardless of CSP type. No difference was observed in mycovirus infections between MAT1-1 and MAT1-2 isolates. DsRNA mycovirus infections in *A. fumigatus* are not related to either CSP or mating type and therefore represent an interesting future therapeutic tool to combat fungal infections.

## Introduction

*Aspergillus fumigatus* is a filamentous fungus that is capable of causing a wide range of diseases in human. Inhaled conidia can cause allergic responses such as Allergic Bronchopulmonary Aspergillosis (ABPA) or allergic *Aspergillus* sinusitis. ABPA affects 7 - 9% of cystic fibroses (CF) patients and 1 – 2% individuals that suffer from asthma [1]–[3]. In immunocompromised patients, *A. fumigatus* can cause invasive aspergillosis of which invasive pulmonary aspergillosis (IPA) is the most common manifestation. Despite adequate antifungal treatment mortality rates remain high, over 50% [4], [5]. Currently, voriconazole is the drug of choice for treatment of invasive aspergillosis, especially in patients with a compromised immune system. However, resistance towards azole antifungal agents is emerging, which may contribute to the dismal outcome of invasive aspergillosis. Therefore, the need for novel therapeutic approaches in the fight against *A. fumigatus* is growing.

Mycoviruses are viruses that selectively infect fungi and are ubiquitous in all major groups of filamentous fungi. Most mycoviruses have segmented double-stranded RNA (dsRNA) genomes packed in a non-enveloped isometric virus-like particle. They usually belong to the virus families *Chrysoviridae*, *Hypoviridea*, *Partitiviridae* and *Totiviridae* and are transmitted by hyphal anastomosis and heterokaryosis. Mycoviruses generally cause cryptic, latent and persistent infections, although some mycoviruses have severe effects on the physiology of the host leading to hypovirulence that is observed as alterations in fungal growth rate, asexual sporulation, fertility and pigmentation [6]–[8]. The induction of hypovirulence led to the use of dsRNA mycoviruses as biological control agents in phytopathogenic fungi [7], [9].

The observation of hypovirulence led to our hypothesis that dsRNA viruses could also be used as ‘biological control agents’ in human fungal infections [6]. Mycoviruses have been observed in some populations of *A. fumigatus* [10], though not in all [11]. Some *A. fumigatus* isolates do seem to show deleterious effect of the infection [12]. Therefore, in order to use dsRNA mycoviruses to control *A. fumigatus* infections it has to be determined if mycoviruses are able to cause infection in all *A. fumigatus* isolates or only in isolates with a certain genetic make-up.

A new typing strategy to discriminate between *A. fumigatus* strains was recently developed. This single locus sequence typing strategy is based on a 12 bp repeat region in the putative cell surface protein (CSP) gene of *A. fumigatus* (AFUA_3G08990) [13], [14]. The DNA sequence variation in the CSP gene has resulted in 18 described CSP types in the entire *A. fumigatus* population so far. CSP typing is a useful first line approach to type *A. fumigatus* isolates and determine the relationship between isolates. For example Camps *et al*. investigated if the trait “azole resistance *A. fumigatus* isolates” was associated with a specific CSP type. It appeared that azole resistance was associated with one specific CSP type [15]. This not only illustrates that CSP typing can distinguish *A. fumigatus* isolates at sub-population level, but also suggests that certain traits such as mycovirus infection may be linked to a certain CSP type.

Recently it was shown that *A. fumigatus* not only reproduces asexually but also sexually via the production of ascospores [16], [17]. The species proved heterothallic and both opposite mating types MAT1-1 and MAT1-2 need to be present for sexual reproduction. Both mating types were found together in the same population [16], [17]. The sexual cycle may be a way to transfer a mycovirus of an infected *A. fumigatus* strain to a non-infected strain, although in *Aspergillus nidulans* the sexual reproduction proved to hinder virus transfer to ascospores [18]. Alternatively direct cytoplasmic transfer between mating partners may open a way for mycovirus transfer. Hence, it is feasible that infection with a mycovirus is associated with a single mating type. In this study, we investigated if the genetic make-up of *A. fumigatus* is correlated to the presence of a mycovirus by determining the CSP and mating type of mycovirus infected and non-infected *A. fumigatus* isolates.

## Materials and Methods

### Collection of *A. fumigatus* isolates

A total of 86 clinical *A. fumigatus* isolates of different patients from the department Microbiology and Infectious Diseases of the Erasmus MC, Rotterdam were used in the study. The clinical isolates were mostly obtained from CF patients and some patients that underwent a lung, heart, liver or kidney transplantation. All isolates were identified as *A. fumigatus* by macroscopic growth on Sabouraud dextrose agar (Difco; Becton & Dickinson) and species identification was confirmed by DNA sequencing of the ITS1-5.8S-ITS2 of the ribosomal operon region [19].

### DNA isolation

To obtain genomic DNA, the isolates were grown on Sabouraud dextrose agar for 4 days at 37°C. The conidia of each isolate were harvested by 0.01% phosphate-buffered saline/TWEEN-20 and genomic DNA from the freshly prepared conidia suspensions was extracted by the total purification DNA kit (Qiagen) as described by the manufacturer.

### CSP typing

Part of the conserved surface protein (CSP) gene was amplified as described previously by Klaassen *et al*.[14]. DNA amplification products were purified and sequenced according to the protocol of the BigDye Terminator v3.1 Cycle sequencing ready reaction kit (Applied Biosystems & life technologies Europe B.V.). Reactions were sequenced on the ABI prism 3100 automated genetic analyzer (Perkin-Elmer Applied Biosystems, Inc.) and analyzed with the Sequencing Analysis (Applied Biosystems, Inc.). To identify the CSP type of an isolate, the DNA sequence was compared for similarities against the proposed nomenclature of 18 CSP sequence types [14].

### Mating type PCR assay

The mating type genotype of each isolate was determined by a multiplex PCR-based mating type test as described previously by Paoletti *et al*. [17].

### Extraction of dsRNA

Spores were grown 48 h in liquid complete medium (CM) [20] in test tubes. The fresh mycelial culture was lyophilized and dsRNA was extracted by a commercial Gaunidium Thiocyanate reagent (TRIZOL, Invitrogen)/Chloroform method previously described by Délye *et al*. [21], with minor modifications: 800 µl TRIZOL reagent was added under sterile conditions to a 1.5 ml microcentrifuge tube containing lyophilized mycelium. The mycelium was than crushed into small particles in the reagent using a sterile micropipette tip. After 5 min incubation on ice, 200 µl chloroform was added to the extract and manually homogenized for 30 sec. After centrifugation for 10 min at 10.000 x g at room temperature, the supernatant was transferred to a new sterile 1.5 ml microcentrifuge tube and 1 µl of RNAseA (4 mg/ml, Promega) was added to the supernatant to digest ssRNA and incubated for 30 min at 37°C. Three hundred µl of NH_4_OAc (2.5 M, Sigma) was subsequently added to the mix and incubated for 30 min at 4°C. The mix was centrifuged and the supernatant transferred to a new 1.5 ml microcentrifuge tube. Next, 600 µl cold isopropanol was added to the supernatant and the mix was incubated at −20°C for at least 30 min. Afterwards the mix was centrifuged for 10 min at 12000 x g and the isopropanol was discarded. One ml of 75% (v/v) ethanol was added to the pellet and the total was centrifuged for 5 min at 4000 x g. After discarding the ethanol, the pellet was dried. Finally, the nucleic acids were resuspendend in 20 µl RNAse-free water (Invitrogen) and incubated overnight at 4°C. One µl ethidium bromide was added to 10 µl of the resuspended nucleic acids in RNAse-free water. Subsequently, 5 µl 1x loading buffer was added to the mix. The dsRNA was made visible by electrophoresis on a 2% agarose RNA gel in DEPC – 0.5 x TBE electrophoresis buffer, performed at 80 mA for 2.5 h and photographed in transmitted ultraviolet light.

### Statistics

Overall percentages were determined by crosstabs (SPSS PASW statistics 17.0.2). The data on dsRNA virus infection frequencies associated with a specific CSP type and mating type were determined using the Fisher exact test (GraphPad Prism version 5.01).

## Results

### The *A. fumigatus* collection could be divided in 11 CSP types

In order to study the genetic diversity in our *A. fumigatus* collection we determined the CSP type in 86 ITS confirmed *A. fumigatus* clinical isolates. Table 1 list the repeat types described by Klaassen *et al*. [14]. We observed 11 different CSP types in our collection: CSP type t01, t02, t03, t04, t05, t06, t08, t09, t011, t013, and a new CSP type that will be referred to as t20 in this study (Table 2). The CSP types t01, t02, t03, and t04 were the most prevalent in our collection.

**Table 1:** Summary of CSP repeat sequences.

Repeat number	Repeat sequence
1	ACTTCTGTCCCG
2	ACTTCTGTCCCA
3	ACTCAAAACGCG
4	ACTTCAATCCCG
5	ACTTTTGTCCCG
6	ACTTCAGTCCCG
7	ACTACTATTGTG
8	ACTTTTCTCCCG
9	ACTTCTGTTCCG

**Table 2:** Overview of the identified CSP types among clinical *A. fumigatus* isolates.

		Codon		Repeat succesion	Codon		Prevelance n(%)
CSP type*	−15	−14	−1		+1	+3	
t01	GTG	GTC	CCG	1-1-1-1-5-3-1-6-3-7	CCA	CCT	22 (25.6)
t02	GTG	GTC	CCG	1-1-2-3-4-5-3-1-6-3-7	CCA	CCT	13 (15.1)
t03	GTG	GTC	CCG	1-2-3-4-6-3-7	CCA	CCT	13 (15.1)
t04	GTG	GTC	CCG/A	1-2-3-4-5-3-1-6-3-7	CCA	CCT	20 (23.3)
t05	GTG	GTC	CCG	1-1-1-3-1-6-3-7	CCA	CCT	6 (7.0)
t06	GTG	GTC	CCG	1-1-1-2-3-4-5-3-1-6-3-7	CCA	CCT	2 (2.3)
t08	GTG	CTC	CCG	1-1-1-2-3-4-5-3-4-5-3-1-6-3-7	CCG	CCTCCT	4 (4.7)
t09	GTG	GTC	CCG	1-1-1-1-1-5-3-1-6-3-7	CCA	CCT	1 (1.2)
t11	GCG	CTC	CCG	1-1-8-3-1-6-3-7	CCA	CCT	3 (3.5)
t13	GTG	CTC	CCG	1-1-2-3-4-5-3-4-5-3-1-6-3-7	CCG	CCTCCT	1 (1.2)
t20^a^	GTG	GTC	CCG	1-1-1-1-1-3-4-5-3-1-6-3-7	CCA	CCT	1 (1.2)

* According to the CSP type nomenclature in Klaassen *et al*. 2009.

a CSP type t20 is not reported among isolates in the previous study of Klaassen *et al*. 2009.

### Double-stranded RNA fragments were obtained in 18.6% of *A. fumigatus* isolates

**Figure 1:**
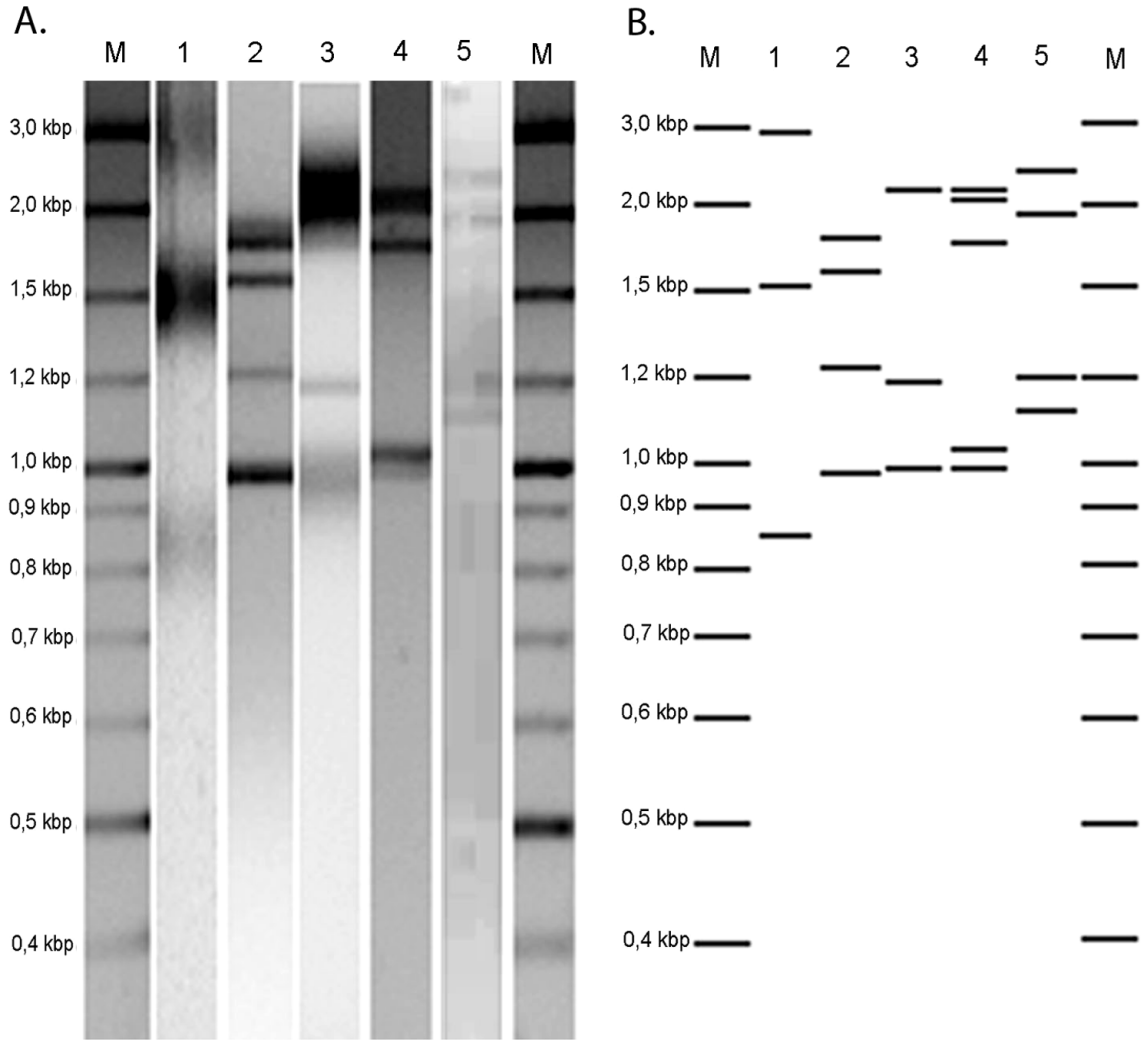
Five putative dsRNA patterns of the 16 mycovirus infected strains. A) Gel electrophoresis of isolated putative dsRNA patterns of *A. fumigatus* isolates, lane 1: dsRNA profiles observed in 11 isolates; lane 2: dsRNA profiles observed in 1 isolate; lane 3: dsRNA profiles observed in 1 isolate; lane 4: dsRNA profiles observed in 2 isolates and lane 5: dsRNA profiles observed in 1 isolate; lane M, DNA size marker. B) The dsRNA profiles displayed in lines.

All 86 clinical isolates were screened for the presence of putative dsRNA viruses, which were detectable as bright and distinct dsRNA bands with gel electrophoresis. We scored the isolates for the presence or absence of the dsRNA segments. The presence of putative dsRNA virus was observed in 16 isolates (18.6%). As seen in figure 1, 5 different dsRNA patterns were detected ranging from 0.87–3 kb in genomic size. Eleven isolates presented 3 dsRNA fragments between 0.87–3 kb and one isolate presented 3 dsRNA fragments between 1.0–2.2 kb. One isolate presented 4 dsRNA fragments between 1.0–1.8 kb and one isolate presented 4 dsRNA fragments between 1.1–2.5 kb. Two isolates contained 5 dsRNA fragments between 1.1–2.2 kb. Since genomes of chrysoviruses and partitiviruses have been observed in many fungal species and can be divided in 2 – 4 segments, we tried a PCR with primer sets designed to generate amplicons of the coding regions of the RNA dependent RNA polymerase (RdRP) and capsid protein of the chrysovirus and partitivirus previously described by Bhatti *et al*. [12]. However, no amplification occurred. Phenotypic differences between infected and non-infected *A. fumigatus* isolates were not observed.

### Infection with dsRNA is not related to a certain CSP type

**Figure 2:**
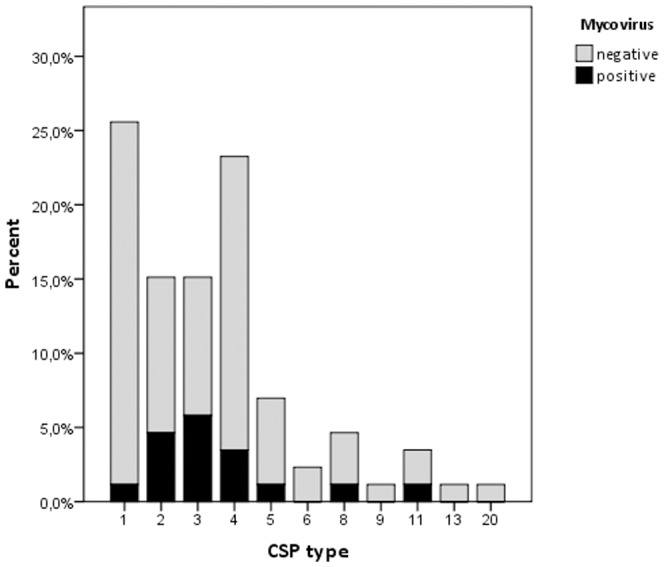
Frequency of CSP types among dsRNA infected and non-infected *A. fumigatus* strains. In 86 *A. fumgiatus* clinical isolates 11 CSP types were detected. DsRNA infection was observed in *A. fumigatus* CSP types t01, t02, t03, t04, t05, t08 and t011. No significant difference was observed between dsRNA infected *A. fumigatus* CSP types and non-infected *A. fumigatus* CSP types (Fisher exact, *p* = 0,58).

To assess if a dsRNA infection in *A. fumigatus* was associated with a specific CSP type, we determined the CSP types of all *A. fumigatus* strains. The dsRNA infection was observed in *A. fumigatus* isolates of the CSP types t01, t02, t03, t04, t05, t08, and t011 (Fig. 2). No significant difference was observed between infected *A. fumigatus* CSP types and non-infected *A. fumigatus* CSP types (Fisher exact, *p* = 0.58), indicating that dsRNA mycovirus infection is not associated with a certain CSP type.

### Double-stranded RNA infections are distributed equally among mating type MAT1-1 and MAT1-2

The frequencies of the dsRNA infected *A. fumigatus* mating types MAT1-1 and MAT1-2 were studied to investigate a correlation between mycovirus infection and mating type. Of our 86 *A. fumigatus* clinical isolates 52.4% contained the MAT1-1 type locus and 47.6% contained the MAT1-2 locus. Among the different mating types dsRNA mycovirus infections were evenly distributed, as 13.9% of mating type MAT1-1 and 20.5% of mating type MAT1-2 was infected (Fisher exact, *p* = 0.40). Furthermore, in principle both mating types were found in all CSP types.

## Discussion

We have shown that dsRNA mycovirus infection of *A. fumigatus* strains is not related to a certain CSP type or mating type. This is an important finding since dsRNA mycoviruses must be able to infect almost all *A. fumigatus* isolates, before they can be regarded as a therapeutic tool for invasive aspergillosis. This is one of the requirements that must be met by dsRNA mycoviruses before they can be regarded as a therapeutic tool for invasive aspergillosis. Given the current limited options to treat potentially lethal invasive *A. fumigatus* disease, the increasing resistance towards the azole class of antifungal agents and the growing need for new therapeutic strategies, the results of this study indicate that dsRNA mycoviruses may indeed be mentioned as potential therapeutic tools.

Based on CSP typing, our collection proved to be of heterogeneous origin and the CSP types found in our collection were comparable with CSP types found in other Dutch collections [14]. In the study of Klaassen *et al*. [14] it was shown that the CSP types t01 to t04 are the most prevalent in the Netherlands and this is confirmed by our study. Similarly, mating types MAT1-1 and MAT1-2 were evenly distributed in our collection which is comparable MAT1-1:MAT1-2 ratio that is observed in worldwide clinical and environmental *A. fumigatus* isolates [17]. This also indicates that our collection and results are representative for many *A. fumigatus* collections world-wide.

To estimate how many isolates were infected with dsRNA mycoviruses, we isolated dsRNA by the TRIZOL/chloroform method previously described by Délye *et al*. [21]. Although the demonstration of large dsRNA bands on electrophoresis gel, is a commonly used technique to demonstrate the presence of mycoviruses in fungi [8], [10], [11], [18], [22], [23], theoretically these dsRNA fragments could be derived from other sources. For example, it has been known that small interfering dsRNA is present in *Aspergillus* species and that this can be detected by isolating dsRNA. Recently, Hammond *et al*.[22] has shown that RNA interference does occur in a mycovirusinfected *A. nidulans* strain. However, the size of this small interfering dsRNA is only 22–24 nucleotides, and since the size of the dsRNA fragments identified here was at least 0.87 kb, we feel that it did not interfere with our interpretation of the results. The observed dsRNA fragment sizes in our study ranged between 0.87 kb and 3 kb which was comparable to the sequenced unipartite and multipartite dsRNA fragment sizes observed from *Aspergillus* isolated mycoviruses belonging to members of the *Chrysoviridae*, *Totiviridae* or *Partitiviridae* [22], [24], [25]. Furthermore, others also demonstrated that the presence of these dsRNA fragments corresponded to the presence of isometric particles by electronic microscopy [8], [26]. Based on these earlier findings, that dsRNA patterns between 3.7– 1220 kb could be classified as mycoviridae, it is most likely that our study observations of comparable dsRNA genomic patterns are indeed to be true putative mycoviruses. We have observed that at least 18.6% of the collection carried a dsRNA mycovirus. Previous studies of a wide range of *Aspergillus* species led to a frequency observation of 0 –13% dsRNA carriage [10]–[12], [27], [28]. Limiting the observations to only *A. fumigatus* isolates, the dsRNA carriage decreases to 0 – 6.9% [10], [11], [27]. Thus the previously reported mycovirus carriage is lower than the carriage reported in our study. The higher frequency in our study cannot be attributed to the genetic make-up of the *Aspergillus* strains in our collection as we found similar CSP types and mating type frequencies as others. However, the observed dsRNA carriage difference could be related to either the thorough detection method used or to the geographic origin of the collection. The dsRNA isolating method that we used was not used by the studies that we compare our results with. This method by Délye *et al*.[21] has been used for isolating dsRNA mycoviruses of the phytopathogenic fungus *Uncinula necator*. With the technique, they also observed higher dsRNA carriage percentages in their European fungal isolates compared with that in North – American fungal isolates. They stated that the origin of the isolates explained the difference in the observed dsRNA frequencies [21]. As this is the first time that the frequency of dsRNA infection in *A. fumigatus* strains is investigated in the Netherlands, the observed difference found in dsRNA carriage in our study compared to others may be due to the geographical origin, or to the sensitivity of the method.

In addition we investigated if the mycovirus observed in our collection could be a chryso- or partitivirus with the primers described in Bhatti *et al*. [10]. However our PCR results were negative. A possible explanation could be that we completely observed different dsRNA patterns of a genomic size compared to the dsRNA patterns that were sequenced as a chrysovirus and partitivirus in the study of Bhatti *et al*. [10] Indeed, the genomic sizes of the dsRNA fragments in our study ranged from 0.87– 3 kb and were smaller in size compared to those in the study in Bhatti *et al*. [10] This might explain our negative PCR results and indicate that the dsRNA that we detected representing members of yet uncharacterized *Chryso*- and *Partitiviridea* or belong to another virus family.

As stated above, an essential finding of our study is that mycoviruses are not limited to a certain CSP or mating type, indicating that mycoviruses are able to infect all *A. fumigatus* isolates. Even though a relatively small number of isolates were used in the study, mycoviruses were found in all CSP types except types 6, 9, 13 and 20. Since the number of *A. fumigatus* strains with these CSP types were only limited to 1 or 2 strains, we believe that the small sample size was the reason for not finding any mycovirus in these groups. However, only if a larger sample size for these CSP types is used, it can be ascertained that mycoviruses are able to infect strains with these CSP types. We therefore conclude that the susceptibility to a mycovirus infection seemed not to be restricted to a certain CSP type. Therefore our study has shown that the first requirement for a mycovirus to use as a therapeutic tool that can be applied to all *A. fumigatus* infected patients. Although CSP typing is highly reproducible and an easy laboratory technique for typing at the sub-population level of *A. fumigatus* isolates, it may have one drawback: CSP typing is not as discriminatory as STRA*f* typing to determine the relation between *Aspergillus* strains [13], [14], [29], [30]. Thus, by using CSP typing we might have missed sub-populations that are naturally resistant to mycovirus infections. If this is the case, it means that the first requirement may not be met. However, this will probably be negligible portion of the *A. fumigatus* making it less likely that this would hinder the development of mycoviruses as therapeutic tools.

The second requirement for mycoviruses as a possible therapeutic tool is that the mycovirus should be able to be transferred from one strain to another. Vertical transfer from an infected strain to asexual conidiospores seems to be generally very efficient, while to sexual ascospores it may be less efficient [18], [28]. Horizontal transfer between two unrelated strains may be aided by the fact mating types occur in approximately equal ratios and strains may be searching for a partner. Heterokaryon incompatibility under the control of heterokaryon incompatibility genes (*het*-genes) is widespread in some *Aspergillus* species and may be blocking vegetative virus transfer [28], however in the case of *A. fumigatus* putative *het*-genes have been identified in the genome sequences while also groups of vegetative compatible clinical isolates have been found [31]. An extracellular phase is so far never found for mycoviruses.

The third important requirement is that mycoviruses should also be able to cause hypovirulence of the fungal host. We did not observe any phenotypic alternations in our dsRNA infected *A. fumigatus* isolates. However, mycoviruses generally belonging to the virus family *Chrysoviridea* or *Partitiviridea* can cause phenotypic alteration and attenuation of the growth of their *A. fumigatus* host [12]. Also in *A. niger*, mycoviruses have been identified with strong fitness effects, with and without phenotypic effects [8]. Intriguingly it has been show that mycoviruses can be viably transmitted between species of the same genus [18], [28], but also between distant/related species [32]. Use of a strongly deleterious mycovirus could potentially be introduced in any *A. fumigatus* background for further spread within an infectious population.

Although we did not sequence the dsRNA mycoviruses present in our study, this would be the next step in the search of suitable mycoviruses to be used as therapeutic tools. Even if these mycoviruses by themselves were not able to cause hypovirulence, by genetically modifying them into a ‘killer-phenotype’ they might potentially help in the eradication of the fungus in the host. This latter step may even be essential to successful use of mycoviruses as therapeutical agents since a recently published paper demonstrated that the fungal burdens in the lungs of immunosuppressed mice were similar when infected with virus-free *A. fumigatus* or *A. fumigatus* containing a weakened chrysovirus [12].

A possible other approach to develop a killer-phenotype mycovirus could be obtained by the use of its specific viral structures as carriers. In plant viruses it has already been shown that viral coat proteins (CP) can function as carrier molecules. By modifying the CP structural properties the viruses could be used as a drug delivery system [33]. This fusion strategy has been applied in several research studies by expressing foreign antigenic epitopes as a novel therapeutic approach for foot and mouth disease, rabies and HIV 1 infection [34], [35]. However, drawbacks of this fusion strategy are the unstable assembly properties of the viral CP to molecules or peptides [33]–[35]. Nonetheless, much more research is needed to establish accepted viable viral CP-molecule delivery systems.

Still, based on the fact that mycoviruses are not restricted to a certain *Aspergillus* CSP and mating type and based on the results achieved with plant viruses, mycovirus therapy remains a promising direction for a novel therapeutic approach for *A. fumigatus* infections in the future.
